# A Highly Nanoporous Nitrogen-Doped Carbon Microfiber Derived from Bioresource as a New Kind of ORR Electrocatalyst

**DOI:** 10.1186/s11671-019-2854-9

**Published:** 2019-01-15

**Authors:** Chaozhong Guo, Yanrong Li, Ya Xu, Qin Xiang, Lingtao Sun, Weizhong Zhang, Wensheng Li, Yujun Si, Zhongli Luo

**Affiliations:** 1 Research Institute for New Materials Technology, School of Materials and Chemical Engineering, Engineering Research Center of New Energy Storage Devices and Applications, Chongqing University of Arts and Sciences, Chongqing, 402160 China; 2 College of Materials Science and Engineering, Chongqing University of Technology, Chongqing, 400054 China; 3 Hubei Collaborative Innovation Center for Advanced Organic Chemical Materials, and Ministry of Education Key Laboratory for the Green Preparation and Application of Functional Materials, School of Materials Science and Engineering, Hubei University, Wuhan, 430062 China; 4 College of Chemistry and Chemical Engineering, Chongqing University, Chongqing, 400044 Shapingba China; 5 School of Resources and Civil Engineering, Wuhan Institute of Technology, Wuhan, 430070 Hubei China; 6 College of Chemistry and Chemical Engineering, Chongqing University of Technology, Chongqing, 400054 China; 7 College of Chemistry and Environmental Engineering, Sichuan University of Science and Engineering, Zigong, 643000 China; 8 College of Basic Medical Sciences, Molecular Medicine and Cancer Research Center, Chongqing Medical University, Chongqing, People’s Republic of China

**Keywords:** Nanoporous carbon, Carbon microfiber, Oxygen reduction reaction, Electrocatalyst, Bamboo-carbon biowaste

## Abstract

Synthesis of metal-free carbon-based electrocatalysts for oxygen reduction reaction (ORR) to replace the conventional platinum-based catalysts has currently become a hot topic of research. This work proposes an activation-assisted carbonization strategy for the fabrication of nitrogen-doped nanoporous carbon microfibers (Me-CFZ-900) with a high BET surface area (~ 929.4 m^2^ g^−1^) via using melamine as a promoter/nitrogen source and bamboo-carbon biowastes as the carbon source with the help of a zinc chloride activator. Electrochemical tests showed that the Me-CFZ-900 material has exhibited excellent ORR electrocatalytic activity and long-term stability, and also displayed a quasi-four-electron ORR pathway in alkaline electrolyte. We also find that the graphitic-N may be the catalytically active site for the ORR, but the formation of planar-N can further help to promote the ORR activity for our catalysts. The results open a new space and provide a new idea to prepare valuable porous nanocarbon materials on the basis of carbonaceous solid wastes for catalysis of a wide range of electrochemical reactions in the future.

**Electronic supplementary material:**

The online version of this article (10.1186/s11671-019-2854-9) contains supplementary material, which is available to authorized users.

## Background

Advanced electrochemical energy systems, such as fuel cells and metal-air batteries, are considered as promising alternatives for traditional fossil fuels [1, 2]. The oxygen reduction reaction (ORR) is an important reaction in those energy technologies, but it suffers from several shortcomings such as high over-potential, sluggish ORR kinetics, and pathway diversity, limiting the improvement of general performance and conversion efficiency [3, 4]. At present, the Pt-based catalysts have been widely employed to enhance the ORR in practical applications, but high cost and restricted resource of metal-Pt hamper the commercialization [5–7]. Thus, the exploration of cheap, active, and stable Pt-free ORR catalysts is significant to rapidly develop clean energy technologies.

To look for some valuable substitutes for metal-Pt catalysts, the doping of heteroatoms into carbon allotropes such as graphene [8], graphdiyne [9], and carbon nanotube [10] is popularly studied owing to their distinctive physical and electronic structures. Although an immense improvement has been carried out on controlled-fabrication of the doped-carbon catalysts, the origin of ORR catalytic activity is still unclear, which becomes a technical bottleneck in this field [11, 12]. Commonly, the enhancement of ORR activity of doped-carbon catalysts can be attributed to charge modulation and broken electroneutrality caused by the heteroatom doping in the carbon framework [13, 14]. Other researches also demonstrated that the ORR activity of the carbon-based catalysts originates from appropriate doping location and configuration [15–17]. Besides, the doping of heteroatoms such as nitrogen can induce carbon surface polarization, which helps to form new nitrogen-containing active sites, thereby favoring adsorption of atoms and ions [18]. Thus, understanding the contribution of N-rich carbon structures is important for clarifying the ORR catalytically active sites, which can also pave a way to directional design ORR-active and stable doped-carbon catalysts.

The use of natural biomass (e.g., soybean [19], silk fibroin [20], kidney bean [21], and hemoglobin [22]) and animal biowastes (e.g., fish scale [23] and animal blood [24]) as a direct precursor or the nitrogen source of catalytically ORR-active sites was thought as an effective pathway to fabricate the doped-carbon catalysts. More recently, Li et al. also synthesized a doped carbon-based ORR catalyst with three-dimensional porous network via using hemin biomaterial as a single-source precursor and using self-assembled sodium chloride crystallines as the template [25]. Jiang et al. [26] converted the biological enzyme of blood centers into Fe–N_*x*_ catalytically active sites for ORR electrocatalysis by the multi-step pyrolysis of blood biowaste. The resulting electrocatalyst shows superior ORR catalytic activity, indicating that the Fe–N_*x*_ structure of heme in blood cells is beneficial in the formation of ORR active centers and therefore can promote the performance of catalysts. These studies may be an aspiration that a new kind of high-performance doped-carbon catalysts can be prepared by appropriately controlling pyrolysis processes and choosing inexpensive biomass materials as the precursors.

Herein, inspired by cheap and easily available biowaste-derived heteroatom-doped carbon for superior ORR performance, we develop a strategy to synthesize a N-doped nanoporous carbon microfiber as a new kind of ORR electrocatalyst (Me-CFZ-900) by pyrolysis of wasted bamboo-carbon tissues with the activation of zinc chloride, combined with the use of melamine as a promoter/nitrogen source. To the best of our knowledge, there are no reports on the design of porous carbon microfibers as an ORR catalyst via facile conversion of bamboo-carbon biowastes until now. We find that the prepared Me-CFZ-900 catalyst has a large number of uniform mesopores with an average pore-diameter of 2.23 nm and a high surface area (~ 929.4 m^2^ g^−1^), which can be beneficial to the mass transportation of O_2_ electrocatalytic reduction. This study opens a new space and provides a new idea to prepare valuable porous nanocarbon materials, which can function as the promising ORR electrocatalysts by further improving pore characteristics and content of active N species.

## Methods

### Synthesis of Carbon-Based ORR Catalysts

**Fig. 1:**
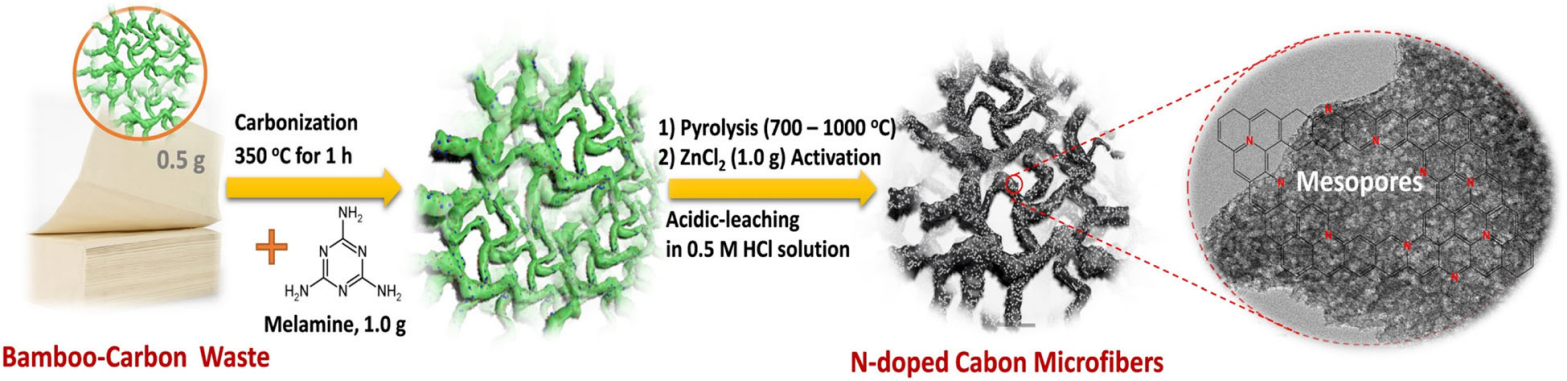
The schematic illustration for synthesis of mesoporous nitrogen-doped carbon microfibers for ORR electrocatalysis via the activation-assisted carbonization of bamboo-carbon biowastes

The nitrogen-doped nanoporous carbon microfibers were prepared via a simple and facile two-step pyrolysis of wasted bamboo-carbon tissues (purchased from Fujian Hengan Group Co. Ltd., China) with the help of zinc chloride activation. Typically, wasted tissues were shredded in a pulper and then carbonized at 350 °C for 1 h in a tube furnace with a heating rate of 20 °C min^−1^ under nitrogen atmosphere to remove some residual organic substances. The obtained carbon microfibers are labeled as the CF350. Subsequently, 0.5 g of CF350, 1.0 g of melamine, and 1.0 g of zinc chloride were uniformly mixed by simple all-solid-state grind for 0.5 h in an agate mortar to obtain a new carbonaceous precursor (Me-CFZ). The Me-CFZ precursor was further heat-treated at in a tubular furnace at 900 °C for 2 h with a heating-rate of 10 °C min^−1^ under the N_2_ atmosphere, resulting in successful synthesis of Me-CFZ-900. The schematic illustration for synthesis of Me-CFZ-900 via an activation-assisted carbonization method is indicated in Fig. 1. To check the effect of the pyrolysis temperatures on the ORR performance of carbon-based catalysts, we also fabricated other Me-CFZ catalysts at different temperatures, which can be marked as Me-CFZ-700, Me-CFZ-800, and Me-CFZ-1000, respectively. As a control, CF-900 and CFZ-900 without the addition of melamine were similarly prepared. All samples were further treated via immersing in a 0.5 mol l^−1^ HCl solution for 2 h before they can be used as an ORR electrocatalyst. For assuring the reproducibility, we prepared all ORR catalysts for three times and their errors can be controlled in the range of 5.0%.

### Physical Characterization

High-resolution scanning electron microscopy (SEM) and transmission electron microscopy (TEM) tests were acquired by Hitachi UHR S4800 (Japan) and FEI Tecnai-G2 F30 instrument with an acceleration voltage of 300 kV, respectively. X-ray photoelectron spectroscopy (XPS) was performed using a Kratos XSAM800 spectrometer. A Micromeritics Analyzer (ASAP 2010) was applied to test N_2_-adsorption/desorption isotherms at 77 K. X-ray diffraction (XRD) analysis was carried out by using a Shimadzu XRD-6000 X-ray diffractometer (Japan) with Cu Ka_1_ radiation (*λ* =1.54178 Ǻ) at 4° min^− 1^. Raman spectroscopy data were recorded with Horiba HR800 Raman system with a laser excitation wavelength of 514.5 nm. XR X-ray was done using a Shimadzu XRD-6000 (*λ* = X-ray diffractometer (Japan) with Cu Ka_1_ radiation ).

### Electrochemical Measurements

Electrocatalytic behavior of the carbon-based ORR catalyst was evaluated on a CHI760E Bipotentiostat (Shanghai Chenhua Instruments Co. Ltd., China). A glass-carbon rotation ring-disk electrode (GC-RRDE, *Φ* = 5 mm, Pine Instrument Co.), a saturated calomel electrode (SCE), and a graphite rod (*Φ* = 0.5 cm) were used as working electrode (WE), reference electrode (RE), and auxiliary electrode (AE), respectively. The fabrication of WE refers to our previous reports [12]. Generally, 10 μl of 10 mg ml^−1^ dispersion was pipetted onto the GC-RRDE surface and naturally dried in the air. The mass loading of carbon-based catalysts and commercial Pt/C catalyst (20 wt% Pt, Aladdin Industrial Co. Ltd.) was controlled to be ~ 600 μg cm^−2^. All potentials (vs. SCE) were transformed into the potentials versus the reversible hydrogen electrode (RHE). Furthermore, electrochemical impedance spectra (EIS) were obtained in the presence of a 1 mmol l^−1^ K_3_[Fe(CN)_6_]/K_4_[Fe(CN)_6_] (mole ratio = 1:1) mixture as a redox probe in 0.1 M KCl solution. In order to sufficiently induce complete peroxide decomposition produced during the test, the ring potential was set at 0.5 V (vs. SCE) as reported elsewhere. The %HO_2_^−^ yield and electron transfer number (*n*) during the ORR were calculated using the following equations [25]:

1 $$ \%{HO}_2^{-}=100\times \frac{2{I}_{\mathrm{r}}/N}{I_{\mathrm{d}}+\left({I}_{\mathrm{r}}/N\right)} $$

2 $$ n=4\times \frac{I_{\mathrm{d}}}{I_{\mathrm{d}}+{I}_{\mathrm{r}}/N} $$

where *I*_d_ is the faradaic current at the disk, *I*_r_ is the faradaic current at the ring, and *N* is the collection efficiency of ring electrode (0.38). *n* was calculated from the Koutecky-Levich equation [27]:

3 $$ 1/{j}_d=1/{j}_k+1/B{\omega}^{1/2} $$

4 $$ B=0.62{nFC}_{\mathrm{O}}{D}_{\mathrm{O}}^{2/3}{\nu}^{-1/6}{\omega}^{1/2} $$

where *F* is the Faraday constant, *C*_O_ is the O_2_ saturation concentration in the electrolyte, *D*_O_ is the O_2_ diffusion coefficient in the electrolyte, *ν* is the kinetic viscosity of the electrolyte, and *ω* is the electrode rotation speed, and 0.62 is a constant when the rotation rate is expressed in rpm.

## Results and Discussion

**Fig. 2:**
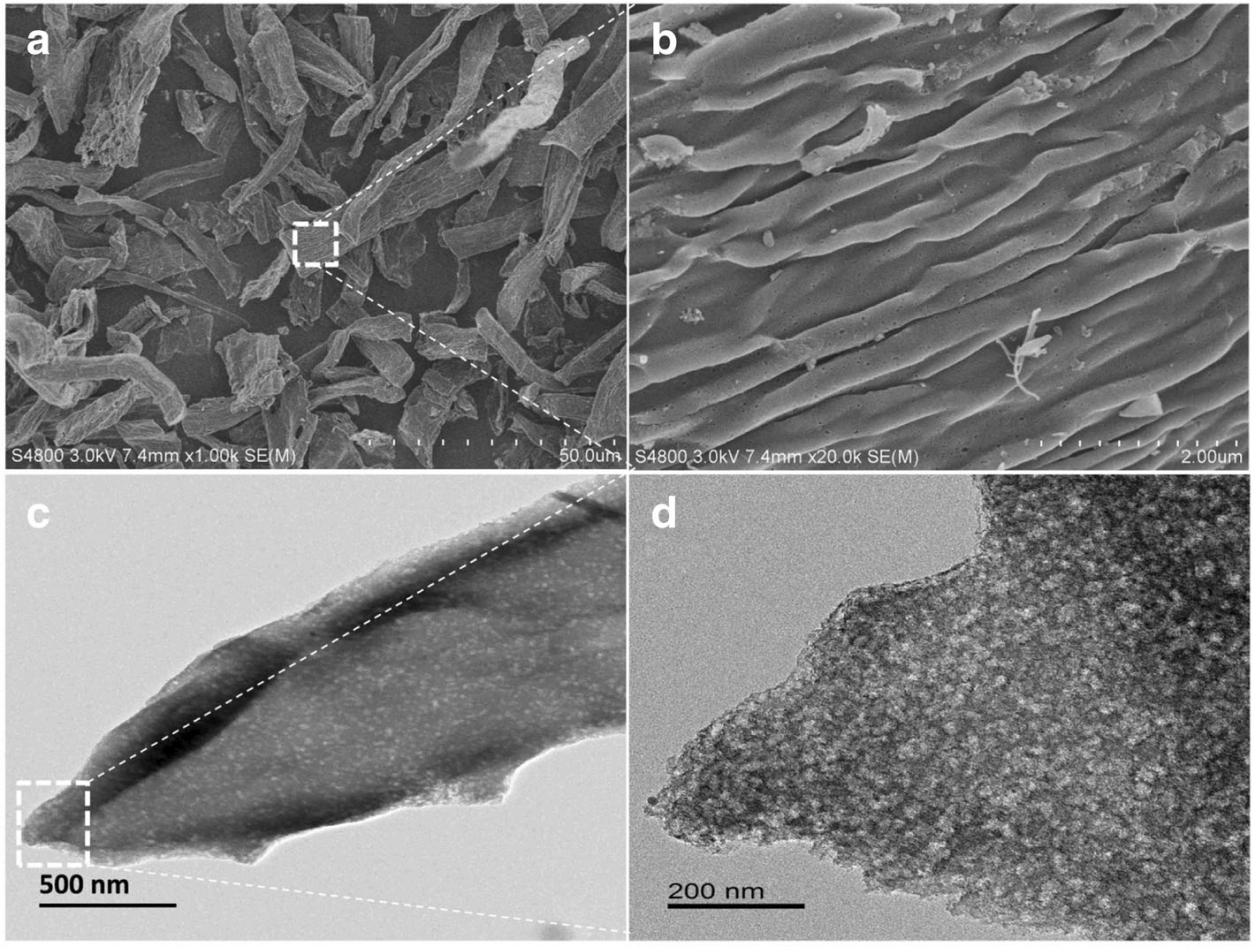
The SEM (**a**, **b**) and TEM (**c**, **d**) images of Me-CFZ-900

Figure 2 shows the SEM and TEM images of the Me-CFZ-900 catalyst. As observed in these SEM images, the Me-CFZ-900 catalyst consists of irregular nitrogen-doped carbon microfibers (Fig. 2a, b). Besides, TEM images of Me-CFZ-900 (Fig. 2c, d) further confirm the results of SEM analysis. The formation of mesopores inside the Me-CFZ-900 catalyst is attributed to the role of zinc chloride activation during high-temperature pyrolysis that induces the rapid dehydration and catalytic dehydroxylation, resulting in the release of hydrogen and oxygen in the form of H_2_O vapor. This activation process can facilitate to produce more mesopores during nitrogen-doping process inside the Me-CFZ-900 catalyst. In addition, some exposed edge defects can be also observed thanks to the N-doping, which is beneficial to promote the ORR catalytic activity.

**Fig. 3:**
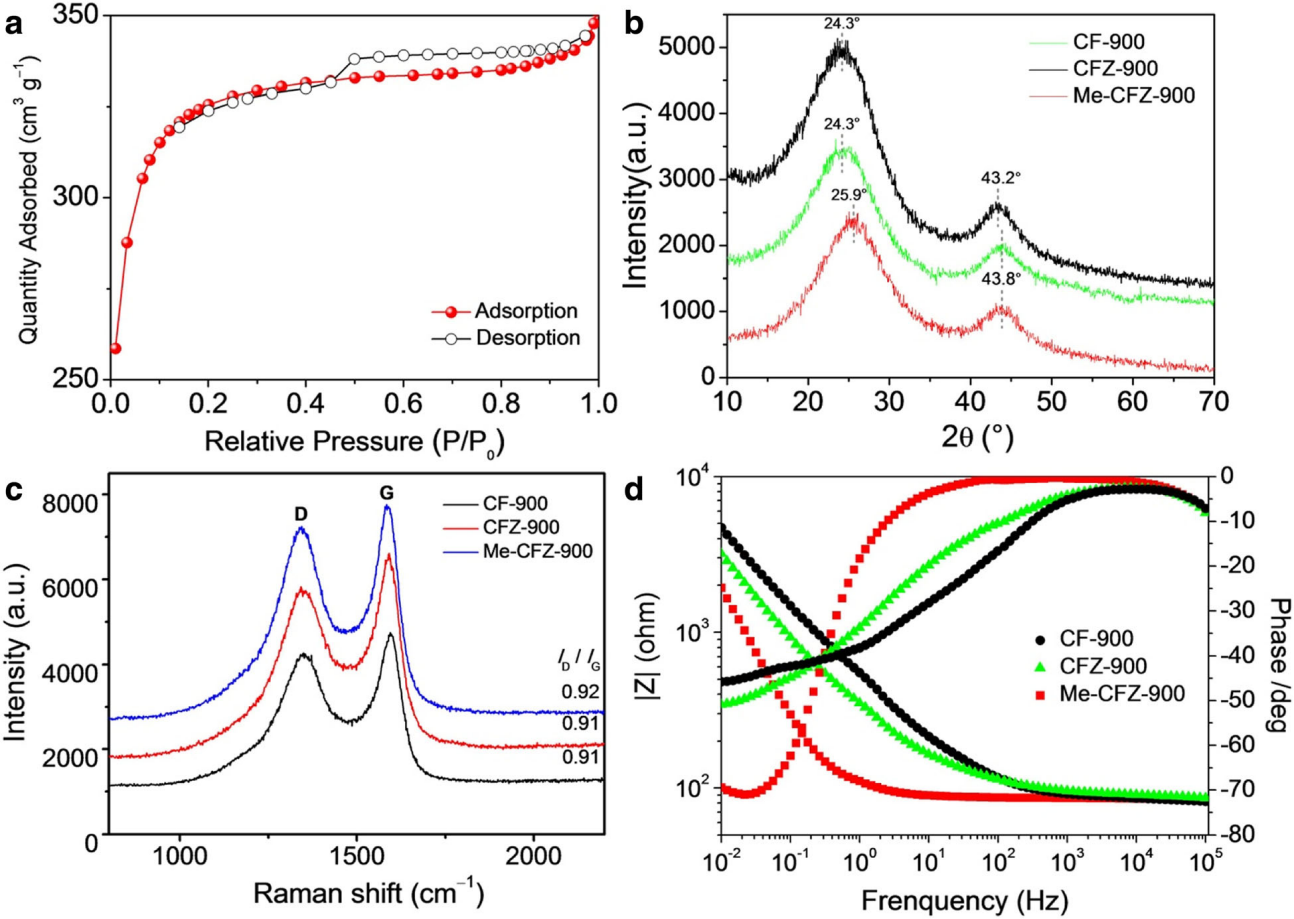
**a** N_2_-sorption isotherms of Me-CFZ-900. **b** XRD patterns of CF-900, CFZ-900, and Me-CFZ-900. **c** Raman spectra of CF-900, CFZ-900, and Me-CFZ-900. **d** Bode spectra of CF-900, CFZ-900, and Me-CFZ-900 under a sine wave of 5.0 mV amplitude in the frequency range of 100 kHz to 10 MHz

Nitrogen adsorption/desorption isotherms were used to examine the Brunauer-Emmett-Teller (BET) specific surface area and pore distribution characteristics, as shown in Fig. 3a and Additional file 1: Figure S1. It is found that a high BET surface area (~ 929.4 m^2^ g^−1^) of Me-CFZ-900 can be ascribed to its rough defect-rich surface and mesoporous characteristics, which is in good agreement with the results of TEM measurements. The BJH pore-size distribution of the Me-CFZ-900 catalyst is indicated in inset of Fig. 3a. The total pore volume of Me-CFZ-900 with an average pore diameter of 2.3 nm is ~ 0.53 cm^3^ g^−1^, but the mesopores mainly focus on the pore diameter of 3.88 nm. These excellent characteristics can be closely related to the enhancement of ORR activity. The carbon structures of different carbon-based ORR catalysts were investigated by X-ray diffraction patterns in Fig. 3b. No crystalline peaks can be observed, except for two carbon planes ((002 )and (101)) located at ~ 24° and ~ 43° respectively, suggesting the amorphous carbon structure [19, 20]. The strong (002) diffraction peak may be mainly attributed to the lattice planes of a typical turbostratic carbon [28]. However, a higher 2-theta of (002) peak and a lower 2-theta of (101) peak for Me-CFZ-900 compared to those for CF-900 and CFZ-900 can be obtained owing to the slight distortion in crystalline regularity along the *a* or *b* direction by the doping of nitrogen atoms in the sp^2^ carbon lattice. Also, all Raman spectra of CF-900, CFZ-900, and Me-CFZ-900 (Fig. 3c) have exhibited two board bands, located at ~ 1345 and ~ 1590 cm^−1^, which are assigned to the disordered sp^3^ carbon (D band) and graphitic sp^2^ carbon (G band), respectively. The intensity ratio (*I*_D_/*I*_G_) of “D” band to “G” band was used to characterize the disordered and graphitic degrees. The corresponding *I*_D_/*I*_G_ for CF-900, CFZ-900, and Me-CFZ-900 are about 0.91, 0.91, and 0.92, respectively. A higher *I*_D_/*I*_G_ ratio of Me-CFZ-900 represents a higher nitrogen-doping efficiency and more defected structure, facilitating to increase the active site density and enhance the ORR electrocatalytic activity. Previous report also proposed that the electrical conductivity of doped-carbon catalysts can be improved by the doping of more nitrogen atoms [25]. For this reason, we further tested the electrical conductivity (EC) of all doped-carbon catalysts by electrochemical impedance spectroscopy (EIS) in 1 mmol l^−1^ K_3_[Fe(CN)_6_]/K_4_[Fe(CN)_6_] (mole ratio = 1:1) probe solution, as displayed in Fig. 3d. The Bode results prove that Me-CFZ-900 offers much lower resistance for mass transport, suggesting better overall conductivity compared to CF-900 and CFZ-900. Furthermore, a higher electrical conductivity of Me-CFZ-900 can help to promote the electron-transportation capacity, resulting in its better ORR activity in alkaline medium.

**Fig. 4:**
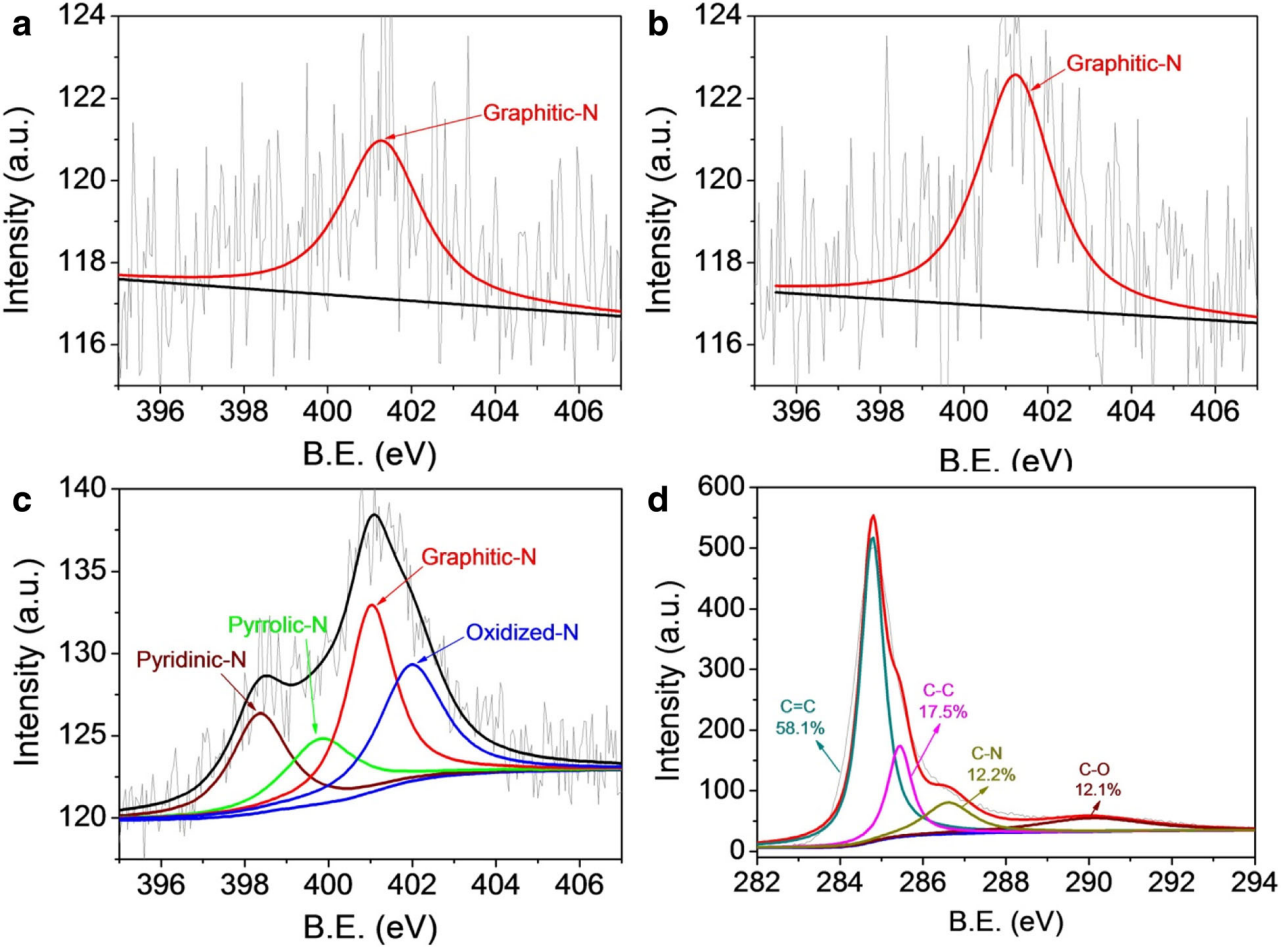
High-resolution N1s XPS spectra of CF-900 (**a**), CFZ-900 (**b**), and Me-CFZ-900 (**c**). **(d**) High-resolution C1s XPS spectra of Me-CFZ-900 (**d**)

The XPS survey data (see Additional file 1: Figure S2) indicate that Me-CFZ-900 is mainly composed of nitrogen, carbon, and oxygen, respectively. The appearance of N1s XPS peak suggests the successful doping of nitrogen into the carbon structure, which is adequately proved by analysis of the C1s peak of Me-CFZ-900. However, the N1s peak of CF-900 and CFZ-900 cannot be observed because of low nitrogen content inside the bamboo-carbon biowaste, as shown in Additional file 1: Figure S1 and Table S1. In addition, the total N content of three doped-N catalysts was determined by surface XPS analysis. The total N content of Me-CFZ-900 is 2.71 at.%, but the total N content is only 0.91 at.% for CF-900 and 0.94 at.% for CFZ-900, respectively. We further check high-resolution N1s XPS spectra of CF-900, CFZ-900, and Me-CFZ-900, as shown in Fig. 4(a–c). The N1s XPS spectra of CF-900 and CFZ-900 can be deconvoluted into one peak with a binding energy (BE) of ~ 401.5 eV, corresponding to the graphitic-N species. However, the high resolution N1s XPS spectrum of Me-CFZ-900 indicates the existence of four types of nitrogen groups: pyridinic-N at 398.3 eV, pyrrolic-N at 398.8 eV, graphitic-N at 401.2 eV, and oxidized-N at 403.4 eV [29–31]. The formation of pyridinic-N and pyrrolic-N is derived from the thermal decomposition of melamine during activation-carbonization process at high-temperatures. In addition, the high-resolution C1s spectra (Fig. 4d and Additional file 1: Figure S3) of CF-900, CFZ-900, and Me-CFZ-900 can be deconvoluted into four peaks at 284.5, 285.9, 287.0, and 293.0 eV, which are assigned to graphitic sp^2^ carbon (C=C), amorphous carbon (C–C), sp^2^ carbon atoms bonded to nitrogen (C–N), and sp^2^ carbon atoms bonded to oxygen (C–O) [32], separately. The proportion of C–N structure increases from 7.8 at.% for CF-900 to 12.2 at. % in Me-CFZ-900, further proving that more nitrogen-atoms have been successfully incorporated into the carbon framework of Me-CFZ-900. Additionally, these results show that the addition of melamine as a promoter and nitrogen source during pyrolysis at 900 °C can affect the total- and doped-N contents and then help to the formation of more active sites inside Me-CFZ-900, facilitating to enhance the ORR catalytic activity during electrochemical tests.

**Fig. 5:**
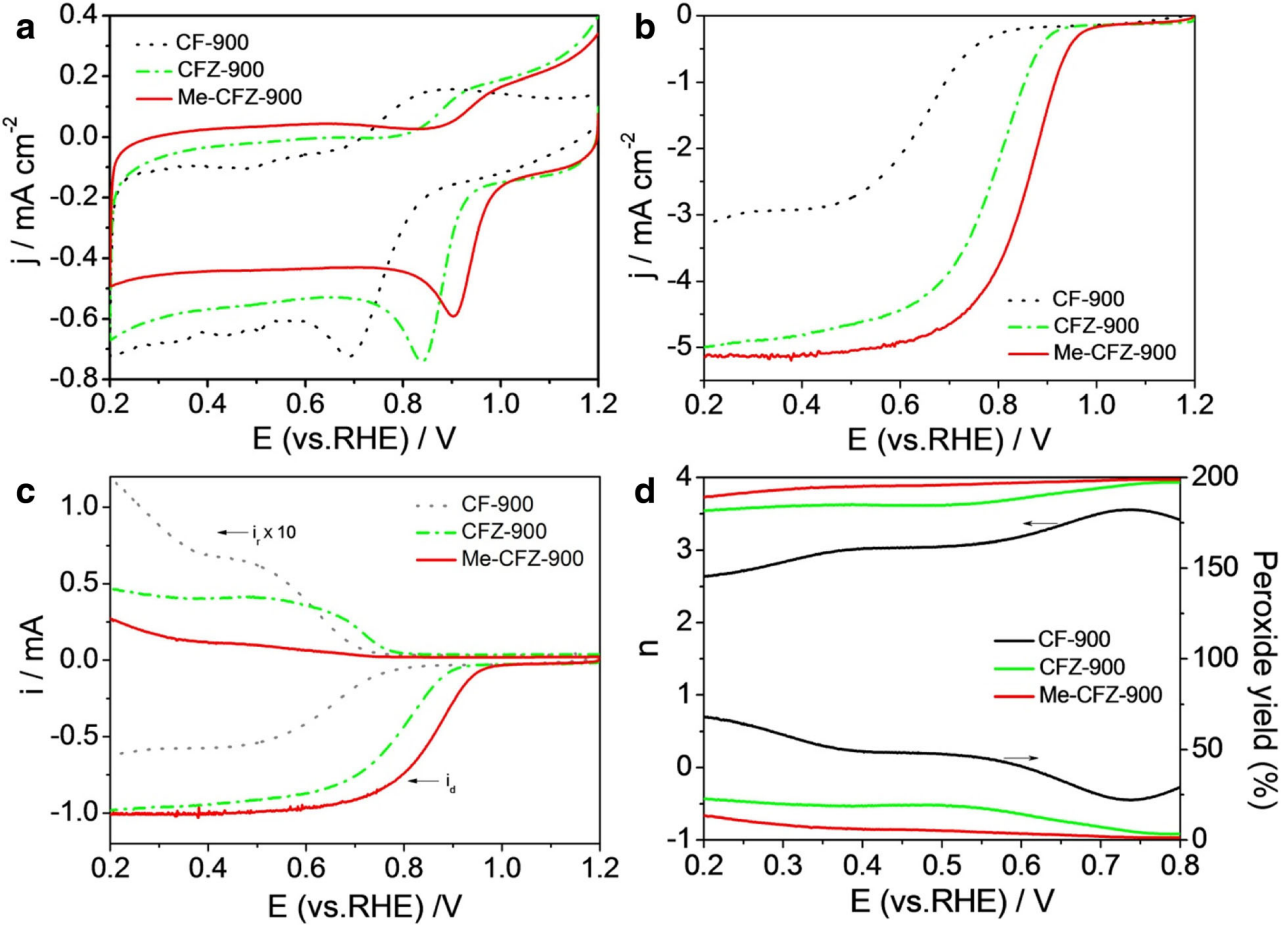
**a** CV curves of CF-900, CFZ-900, and Me-CFZ-900 in O_2_-saturated 0.1 mol l^−1^ KOH solution. **b** LSV curves for ORR of CF-900, CFZ-900, and Me-CFZ-900 and 20 wt% Pt/C in O_2_-saturated 0.1 M KOH solution at a rotation speed of 1600 rpm. **c** Disk and ring currents obtained with LSV on RRDE for CF-900, CFZ-900, and Me-CFZ-900 in O_2_-saturated 0.1 mol l^−1^ KOH solution. **d** The corresponding electron transfer number and H_2_O_2_ yield from **c**

To evaluate the ORR electrocatalytic activity, three carbon-based ORR catalysts were respectively coated on the GC-RRDE surface and further tested by cyclic voltammetry (CV) and linear sweep voltammetry (LSV) in O_2_-saturated 0.1 mol l^−1^ KOH solution. The electrochemical results on the ORR activity are indicated in Fig. 5a. It can be found that all CV curves of CF-900, CFZ-900, and Me-CFZ-900 in O_2_-saturated electrolyte display clear ORR peaks with peak potentials of 0.69, 0.84, and 0.91 V versus RHE, respectively. It is found that the ORR activity for three carbon catalysts follows the order of Me-CFZ-900 > CFZ-900 > CF-900. Furthermore, LSV curves (Fig. 5b) recorded in O_2_-saturated KOH solution were obtained at a rotation rate of 1600 rpm to further understand the catalytic activities of CF-900, CFZ-900, and Me-CFZ-900. The CFZ-900-catalyzed electrode displays better ORR activity with a half-wave potential (*E*_1/2_) of 0.78 V compared to the CF-900-catalyzed electrode with an *E*_1/2_ of 0.65 V versus RHE. Besides, a higher *E*_1/2_ of ~ 0.86 V and larger limited current density at given potentials can be obtained on the Me-CFZ-900-catalyzed electrode, which is comparable to those of the commercial Pt/C (20 wt%) catalyst (see Additional file 1: Figure S4) and other carbon catalysts reported in the literature (see Additional file 1: Table S2). These results are in good accordance with the results of CV measurements, further showing the excellent ORR activity of Me-CFZ-900. It is suggested that the zinc chloride activation and the addition of nitrogen source can improve the ORR catalytic activity due to the generation of mesoporous structures and the improvement of N-doping efficiency during pyrolysis process.

RRDE measurements were carried out to get insights into the ORR kinetics of carbon-based catalysts, as shown in Fig. 5c. Besides, based on the RRDE data, the corresponding electron number transferred (*n*) and peroxide species (H_2_O_2_%) produced during ORR are calculated via using Eqs. (1) and (2), respectively. The calculation results are displayed in Fig. 5d. The H_2_O_2_ yield (< 14.0%) and electron transfer number (3.45–3.95) on Me-CFZ-900 can be found in the potential range of 0.2–0.8 V versus RHE, which indicates a quasi-four-electron pathway for ORR process being similar to the ORR kinetics of commercial Pt/C catalyst (Additional file 1: Figure S3). Compared to the Me-CFZ-900, higher H_2_O_2_ yield and smaller electron transfer number can be observed on both CF-900 and CFZ-900 in the same potential range. However, the H_2_O_2_ yield on CFZ-900 is higher than that on Me-CFZ-900, but the electron transfer number on CFZ-900 is similar to that on Me-CFZ-900, also suggesting a quasi-four-electron pathway for ORR process. Unfortunately, CF-900 has exhibited the lowest electron transfer number (2.64–3.56) and highest H_2_O_2_ yield (22.2–68.2%), implying that the ORR catalyzed by CF-900 mainly follows a mixed pathway of two-electron and four-electron processes. These results prove that the carbon catalysts prepared by the zinc chloride activation have exhibited higher ORR catalytic efficiency and electrocatalytic performance with or without the addition of melamine. Combined with XPS analysis and ORR activity data, we find that only graphitic-N species can exist in CF-900 and CFZ-900 but exhibits ORR catalytic activity, which proves that the graphitic-N can be one of electrocatalytically active sites contributing to the ORR electrocatalysis. It is remarkable that the addition of melamine into the precursor can promote the formation of pyridinic-N and pyrrolic-N species with planar structures, which can be responsible for the ORR activity enhancement, supported by the previously reported results [29]. Besides, the excellent ORR performance of Me-CFZ-900 can be also ascribed to other aspects: (1) high BET surface area and mesoporous structure of Me-CFZ-900 can facilitate the adsorption and transportation of oxygen molecule and the exposure of more active sites; (2) higher electrical conductivity of Me-CFZ-900 can effectively boost the electron transportation of ORR process; and (3) more N atoms are incorporated into the carbon structure of Me-CFZ-900, which can produce more nitrogen-rich defected structures and active sites. Therefore, controlled synthesis of high contents of planar and graphitic nitrogen species is essential to produce the active carbon-based catalysts for ORR, but further improvement of electrical conductivity, nitrogen-doping efficiency, and mesoporous characteristics is the key issue to enhance the ORR catalytic activity.

**Fig. 6:**
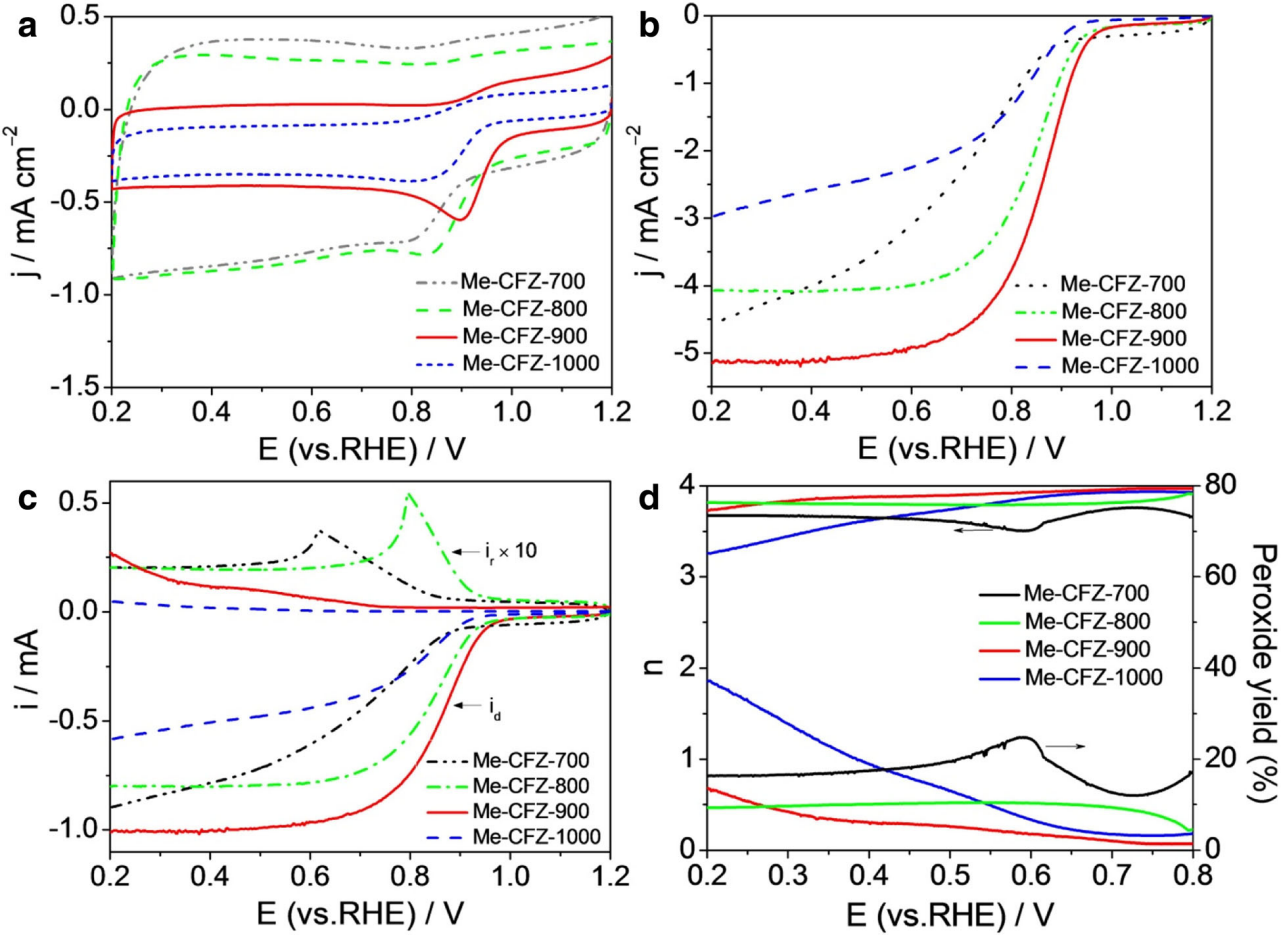
**a** CV and **b** LSV curves of Me-CFZ-700, Me-CFZ-800, Me-CFZ-900, and Me-CFZ-1000 in O_2_-saturated 0.1 mol l^−1^ KOH solution. **c** Disk and ring currents obtained with LSVs on RRDE for Me-CFZ-700, Me-CFZ-800, Me-CFZ-900, and Me-CFZ-1000 in O_2_-saturated 0.1 mol l^−1^ KOH solution. **d** The corresponding electron transfer number and H_2_O_2_ yield from **c**

To better understand the effect of the pyrolysis temperatures on the ORR activity, we also prepared another three catalysts such as Me-CFZ-700, Me-CFZ-800, and Me-CFZ-1000 by the same pyrolysis procedure. In Fig. 6a, all prepared catalysts have exhibited an obvious ORR peak but Me-CFZ-900 has the largest peak current and the most positive peak potential. LSV curves recorded at a rotation speed of 1600 rpm further suggest that the Me-CFZ-900 has exhibited more positive onset and half-wave potentials for ORR compared other catalysts (Fig. 6b). Obviously, the ORR catalytic activity of our prepared catalysts is strongly dependent on the pyrolysis temperature. The optimal temperature is 900 °C for our system, as higher or lower will still yield inferior ORR electrocatalytic activity. It may be owing to the density of active sites and porous characteristics inside catalysts controlled by the pyrolysis temperature. To further understand the ORR kinetics behavior of different carbon-based catalysts, we also perform the RRDE tests to monitor the H_2_O_2_ yield and electron transfer number (Fig. 6c, d). It can be seen that the ring current (*i*_r_) of Me-CFZ-900 is obviously lower than that of other catalysts in the potential range of 0.2–0.8 V, resulting in the highest electron transfer number and the lowest H_2_O_2_ yield on Me-CFZ-900 based on the RRDE data. These results further confirm that the best ORR activity of prepared catalysts can be obtained at 900 °C.

**Fig. 7:**
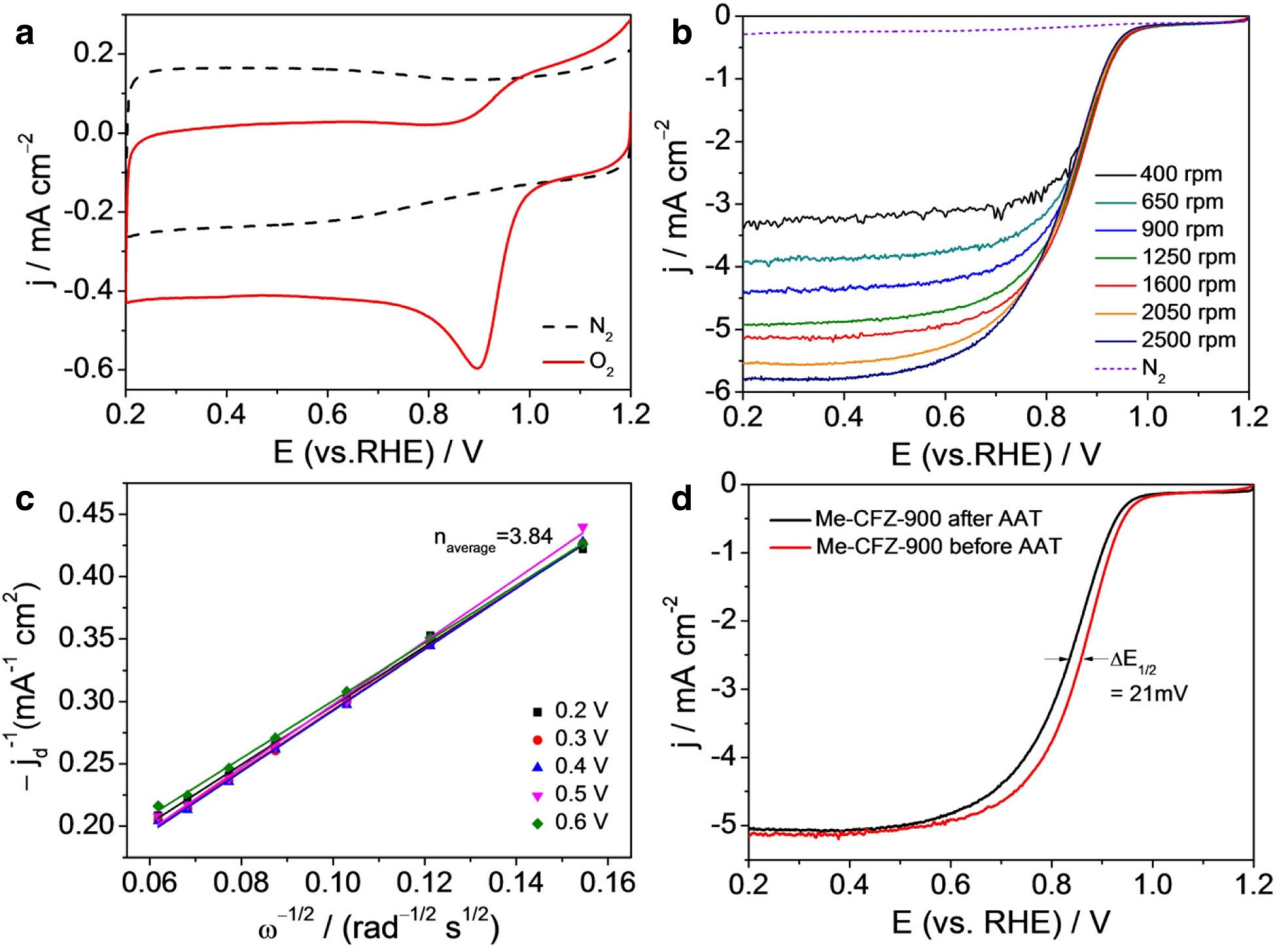
**a** CV curves of Me-CFZ-900 in 0.1 mol l^−1^ KOH solution saturated by N_2_ versus O_2_. **b** LSV curves of Me-CFZ-900 in O_2_-saturated 0.1 mol l^−1^ KOH solution at different rotation speeds (400–3600 rpm). **c** Koutecky-Levich plots of *j*_*d*_^−1^ vs. *ω*^−1/2^ obtained from **b** at given potentials. **d** LSV curves of Me-CFZ-900 in O_2_-saturated 0.1 mol l^−1^ KOH electrolytes before and after AAT test

The ORR catalysis behavior of Me-CFZ-900 was further evaluated by the CV curves and LSV curves in N_2_ versus O_2_-saturated 0.1 mol l^−1^ KOH solutions (Fig. 7). In N_2_-saturated electrolyte, except for a clear capacitive CV, no visible peak can be observed in Fig. 7a, indicating that it is featureless. On the contrary, when the CV test is performed in O_2_-saturated electrolyte, a well-defined ORR peak at ~ 0.90 V is obtained. Above results qualitatively suggest an ORR electrocatalytic activity of Me-CFZ-900 with an ORR onset potential of ~ 1.0 V approaching to the commercial Pt/C catalyst (Additional file 1: Figure S4). Furthermore, to better reveal the ORR process of Me-CFZ-900, RDE measurements were performed at a scanning rate of 5 mV s^−1^ with different rotation rates (400–2500 rpm), as shown in Fig. 7b. The limiting diffusion current density increases with increasing of the rotation speed, demonstrating that the current is kinetically controlled. The Koutecky-Levich plots (*j*^−1^ vs. *ω*^−1/2^) obtained at 0.2–0.6 V show good linearity and near parallelism (or overlap) (Fig. 7a), indicating similar electron transfer numbers for ORR at five potentials (0.2–0.6 V). The average electron transfer number is calculated to be ~ 3.84 from the slope of Koutecky-Levich plots via using Eqs. (3) and (4), further confirming that the ORR on Me-CFZ-900 follows a four-electron reaction pathway, being similar to the Pt/C catalyst [33]. This result is in good agreement with the RRDE tested results. In addition, the stability for ORR electrocatalysis is one of the major concerns in current alkaline fuel cell technology. For this purpose, the long-term stability of Me-CFZ-900 was measured by an accelerated aging testing (AAT) in O_2_-saturated 0.1 M KOH solution. Before LSV tests for ORR catalysis again, the Me-CFZ-900 catalyst suffers from continuous CV measurements between 0.2 and 1.2 V vs. RHE for 5000 cycles at a scan rate of 200 mV s^−1^. As shown in Fig. 7d, LSV curves of Me-CFZ-900 exhibit an only ~ 21-mV negative shift in half-wave potential and about 2.0% decline in limited diffusion current density, but no noticeable reduction in ORR onset potential is observed. Our previous reports show that the commercial Pt/C catalyst commonly shows a ~ 50-mV negative shift in ORR half-wave potential after CV test for 5000 cycles. These electrochemical results suggest a promising long-term stability of Me-CFZ-900, which is obviously superior to the commercial Pt/C catalyst (Additional file 1: Figure S5). In summary, we can find that the Me-CFZ-900 catalyst prepared in this work is a quite promising candidate for Pt-based ORR catalysts in alkaline medium.

## Conclusions

In summary, we develop a new method to prepare nanoporous N-doped carbon microfibers (Me-CFZ-900) derived from bamboo-carbon biowastes for the electrocatalysis of oxygen reduction reaction in alkaline media. The as-prepared Me-CFZ-900 catalyst exhibits the ORR electrocatalytic activity with a half-wave potential of ~ 0.86 V and a peak potential of ~ 0.91 V. The peroxide yield less than 14% and the average electron transfer number of 3.84 are obtained on Me-CFZ-900, further showing a quasi-four-electron reaction pathway. An only 21 mV negative shift in half-wave potential and 2.0% decline in the limited current density are observed on Me-CFZ-900 after doing the accelerated aging test. Furthermore, high BET surface area (929.4 m^2^ g^−1^) and mesoporous structure of Me-CFZ-900 can facilitate the adsorption and transportation of oxygen molecule. This work can help the researchers to build the high-performance carbon-based ORR electrocatalyst derived from biomass wastes and to understand the origin of the ORR electrocatalytic activity.

## Additional File

Additional file 1: **Figure S1.** The BJH pore-size distribution of Me-CFZ-900. **Figure S2.** XPS survey data of CF-900, CFZ-900, and Me-CFZ-900. **Figure S3.** C1s XPS spectra of CF-900 and CFZ-900. **Figure S4.** (a) LSV curves for ORR of Me-CFZ-900 and 20 wt% Pt/C catalyst; (b) The electron transfer number and H_2_O_2_ yield of Me-CFZ-900 and 20 wt% Pt/C catalyst. **Figure S5.** LSV curves for ORR of 20 wt% Pt/C catalyst before and after AAT in O2-saturated 0.1 M KOH solution. **Table S1.** The contents of nitrogen, carbon, and oxygen inside the prepared catalysts detected by elemental analysis. **Table S2.** The ORR catalytic activity data for Me-CFZ-900, other carbon or biowaste-derived catalysts reported in the literature. (DOCX 301 kb)

## Abbreviations

AAT: Accelerated aging test
AE: Auxiliary electrode
BET: Brunauer-Emmett-Teller
CF: Carbon microfibers
CV: Cyclic voltammetry
*E*_1/2_: Half-wave potential
*E*_ORR_: Onset potential
*E*_p_: Peak potential
FE-SEM: Field-emission scanning electron microscopy
GC: Glassy carbon
HR-TEM: High-resolution transmission electron microscopy
LSV: Linear sweep voltammetry
Me-CFZ-900: Nitrogen-doped porous carbon microfibers
ORR: Oxygen reduction reaction
Pt/C: Platinum/carbon catalyst
RDE: Rotation disk electrode
RE: Reference electrode
RHE: Reversible hydrogen electrode
RRDE: Rotation ring-disk electrode
SCE: Saturated calomel electrode
WE: Working electrode
XPS: X-ray photoelectron spectroscopy
