# Supplementary material for: A Highly Nanoporous Nitrogen-Doped Carbon Microfiber Derived from Bioresource as a New Kind of ORR Electrocatalyst
Source: Nanoscale Res Lett. 2019 Jan 15;14:22. doi: 10.1186/s11671-019-2854-9 (PMC6333595; doi:10.1186/s11671-019-2854-9)
Supplement: Supplementary file 1 — Figure S1. The BJH pore-size distribution of Me-CFZ-900. Figure S2. XPS survey data of CF-900, CFZ-900, and Me-CFZ-900. Figure S3. C1s XPS spectra of CF-900 and CFZ-900. Figure S4. (a) LSV curves for ORR of Me-CFZ-900 and 20 wt% Pt/C catalyst; (b) The electron transfer number and H2O2 yield of Me-CFZ-900 and 20 wt% Pt/C catalyst. Figure S5. LSV curves for ORR of 20 wt% Pt/C catalyst before and after AAT in O2-saturated 0.1 M KOH solution. Table S1. The contents of nitrogen, carbon, and oxygen inside the prepared catalysts detected by elemental analysis. Table S2. The ORR catalytic activity data for Me-CFZ-900, other carbon or biowaste-derived catalysts reported in the literature. (DOCX 301 kb) [file 11671_2019_2854_MOESM1_ESM.docx]

**Electronic Supporting Information**

**Highly Nanoporous N-Doped Carbon Microfibers Derived from Bioresouce as a New Kind of ORR Electrocatalyst**

Chaozhong Guo^a†*^, Yanrong Li^c†^, Ya Xu^b†^, Qin Xiang^d†^, Lingtao Sun^a^*, Weizhong Zhang^e^, Wensheng Li^c^, Yujun Si^f*^

^a^Research Institute for New Materials Technology, School of Materials and Chemical Engineering, Engineering Research Center of New Energy Storage Devices and Applications, Chongqing University of Arts and Sciences, Chongqing 402160, China

^b^Hubei Collaborative Innovation Center for Advanced Organic Chemical Materials, and Ministry of Education Key Laboratory for the Green Preparation and Application of Functional Materials, School of Materials science and engineering, Hubei University, Wuhan 430062, P.R. China

^c^College of Materials Science and Engineering, College of Chemistry and Chemical Engineering, Chongqing University of Technology, Chongqing 400054, China

^d^ College of Chemistry and Chemical Engineering, Chongqing University, Chongqing 400044, Shapingba, China

^e^ School of Resources and Civil Engineering, Wuhan Institute of Technology, Wuhan 430070, Hubei, China

^f^College of Chemistry and Environmental Engineering, Sichuan University of Science and Engineering, Zigong, 643000, China

^†^These authors equally contributed to this work, and they are considered as the co-first author.

*Corresponding authors. E-mail: [guochaozhong1987@163.com](mailto:guochaozhong1987@163.com) (C. Guo); 595118563@qq.com (L. Sun); syj08448@163.com (Y. Si)

E-mail: [guochaozhong1987@163.com](mailto:guochaozhong1987@163.com) (Chaozhong Guo); [1793474272@qq.com](mailto:1793474272@qq.com) (Yanrong Li); [1328627836@qq.com](mailto:1328627836@qq.com) (Ya Xu); [657685852@qq.com](mailto:657685852@qq.com) (Qin Xiang); [595118563@qq.com](mailto:595118563@qq.com) (Lingtao Sun); [419952520@qq.com](mailto:419952520@qq.com) (Weizhong Zhang); [411587543@qq.com](mailto:411587543@qq.com) (Wensheng Li); syj08448@163.com (Y. Si)

**Figure S1.** The BJH pore-size distribution of Me-CFZ-900.

**Figure S2**. XPS survey data of CF-900, CFZ-900 and Me-CFZ-900.

**Figure S3**. C1s XPS spectra of CF-900 and CFZ-900.

**Figure S4**. (a) LSV curves for ORR of Me-CFZ-900 and 20 wt.% Pt/C catalyst; (b) The electron transfer number and H_2_O_2_ yield of Me-CFZ-900 and 20 wt.% Pt/C catalyst.

**Figure S5**. LSV curves for ORR of 20 wt.% Pt/C catalyst before and after AAT in O2-saturated 0.1 M KOH solution.

**Table S1.** The contents of nitrogen, carbon and oxygen insides the prepared catalysts detected by elemental analysis.

| **Sample** | **Nitrogen (at.%)** | **Carbon (at.%)** | **Oxygen (at.%)** |
| --- | --- | --- | --- |
| CF-900 | 0.91 | 93.24 | 5.85 |
| CFZ-900 | 0.94 | 94.35 | 4.71 |
| Me-CFZ-900 | 2.71 | 95.76 | 2.13 |

**Table S2**. The ORR catalytic activity data for Me-CFZ-900, other carbon or biowaste-derived catalysts reported in the literature.

| **Samples** | ***E*_ORR_** | **E_1/2_** | **n** | **Current density** | **Ref.** |
| --- | --- | --- | --- | --- | --- |
| Fe/C-SOYB-A | 0.83 V *vs.*RHE | 0.66 V *vs.*RHE | 3.1 | 1.3 mA cm^–2^ @ + 0.65 V *vs.*RHE | [19] |
| BP350C1000 | 0.90 V *vs.*RHE | 0.78 V *vs.*RHE | 3.5 | 1.0 mA cm^–2^ @ + 0.65 V *vs.*RHE | [24] |
| N-Graphene | 0.31 V *vs.*SHE | 0.35 V *vs.*SHE | 3.6 | 3.0 mA cm^–2^ @ –1.0 V *vs.*SHE | [34] |
| Co-N-C(900) | 0.85 V *vs.*RHE | 0.80 V *vs.*RHE | 3.9 | 5.5 mA cm^–2^ @ + 0.65 V *vs.*RHE | [35] |
| CoN/C-600 | 0.91 V *vs.*RHE | 0.85 V *vs.*RHE | 3.8 | 5.7 mA cm^–2^ @ + 0.55 V *vs.*RHE | [36] |
| N-CNT(800) | 0.91 V *vs.*RHE | 0.70 V *vs.*RHE | 3.7 | 2.6 mA cm^–2^ @ +0.65 V *vs.*RHE | [37] |
| GO flakes | –0.21 V *vs.*Ag/AgCl | --- | 1.9 | 3.7 mA cm^–2^ @ –1.0 V *vs.*Ag/AgCl | [38] |
| Co/N/C-900 | 0.035 V *vs.*Hg/HgO | --- | 3.8 | 4.3 mA cm^–2^ @ –0.4 V *vs.* Hg/HgO | [39] |
| Me-CFZ-900 | 1.0 V *vs.*RHE | 0.86 V vs.RHE | 3.8 | 5.2 mA cm^–2^ @ + 0.50 V *vs.*RHE | This work |

[34] D. Geng, Y. Chen, Y. Chen, Y. Li, R. Li, X. Sun, S. Ye and S. Knight, *Energy Environ. Sci.*, 4 (2011) 760.

[35] C. Guo, Y. Wu1, Z. Li, W. Liao, L. Sun, C. Wang, B. Wen, Y. Li and C. Chen, *Nanoscale Res. Lett.*, 12 (2017) 144.

[36] S. Chao and M. Jiang, *Int. J. Hydrogen Energy*, 41 (2016) 12995.

[37] J. Zheng, C. Guo, C. Chen, M. Fan, J. Gong, Y. Zhang, T. Zhao, Y. Sun, X. Xu, M. Li, R. Wang, Z. Luo, C. Chen, *Electrochim Acta*, 168 (2015) 386.

[38] J. Liu, H. Yang, S. Zhen, C. Poh, A. Chaurasia, J. Luo, X. Wu, E. Yeow, N. Sahoo, J. Lin and Z. Shen, *RSC adv.*, 3 (2013) 11745.

[39] H. Wu, C. Guo, J. Li, Z. Ma, Q. Feng and C. Chen, *Int. J. Hydrogen Energ.*, 41 (2016) 20494.
